# Multimode fiber endoscopes for computational brain imaging

**DOI:** 10.1117/1.NPh.11.S1.S11509

**Published:** 2024-03-06

**Authors:** Lyubov V. Amitonova

**Affiliations:** a Vrije Universiteit Amsterdam, Department of Physics and Astronomy, Amsterdam, The Netherlands; b Advanced Research Center for Nanolithography, Amsterdam, The Netherlands

**Keywords:** neuroimaging, optical microscopy, endoscopy, computational imaging, multimode fibers

## Abstract

Advances in imaging tools have always been a pivotal driver for new discoveries in neuroscience. An ability to visualize neurons and subcellular structures deep within the brain of a freely behaving animal is integral to our understanding of the relationship between neural activity and higher cognitive functions. However, fast high-resolution imaging is limited to sub-surface brain regions and generally requires head fixation of the animal under the microscope. Developing new approaches to address these challenges is critical. The last decades have seen rapid progress in minimally invasive endo-microscopy techniques based on bare optical fibers. A single multimode fiber can be used to penetrate deep into the brain without causing significant damage to the overlying structures and provide high-resolution imaging. Here, we discuss how the full potential of high-speed super-resolution fiber endoscopy can be realized by a holistic approach that combines fiber optics, light shaping, and advanced computational algorithms. The recent progress opens up new avenues for minimally invasive deep brain studies in freely behaving mice.

## 1 Introduction

The field of neurophotonics is built upon continuous technological advances in light microscopy. Ever since Antoni van Leeuwenhoek observed cells under an early microscope, optical microscopy remains the key instrument in neuroscience.^1^ Technological breakthroughs in optical imaging are constantly changing the way neural circuits can be examined and visualized.^2^ Modern benchtop systems provide high-resolution multifunctional imaging but require head fixation of the animal under the microscope objective,^3^ which is unfortunately incompatible with many behavioral studies. Miniaturized microscopes have been developed to enable measurements in freely moving animals.^4^^,^^5^ However, miniscopes as well as many other state-of-the-art optical techniques work well only at surface or sub-surface areas up to a few hundred micrometers in depth,^6^ but neurophotonics research requires high-resolution images *in vivo* in deeper layers of the brain.^7^ Gradient-index (GRIN) lenses are used for deep imaging but due to the relatively large size the implantation includes tissue removal.^8^ Optical imaging at truly unlimited depths has been enabled by minimally invasive endo-microscopy based on bare optical fibers.^9^ The most straightforward approach of lensless fiber-imaging is to use fiber bundles where each fiber transmits one pixel of an image.^10^^–^^12^ However, the spatial resolution is relatively low due to large core-to-core spacing.

To summarize, the problem of understanding the relationship between neural activity in deep brain structures and unrestrained behavior remains unsolved. One of the most promising research directions addressing this challenge is novel imaging approaches based on a multimode fiber (MMF).^13^ The full potential of minimally invasive MMF endoscopy for neuroimaging—ultimate performance when used for deep-tissue imaging—can only be realized by combining optimal probes, advanced light control, and computational post-processing algorithms.

## 2 Multimode Fiber Endoscopes

**Fig. 1:**
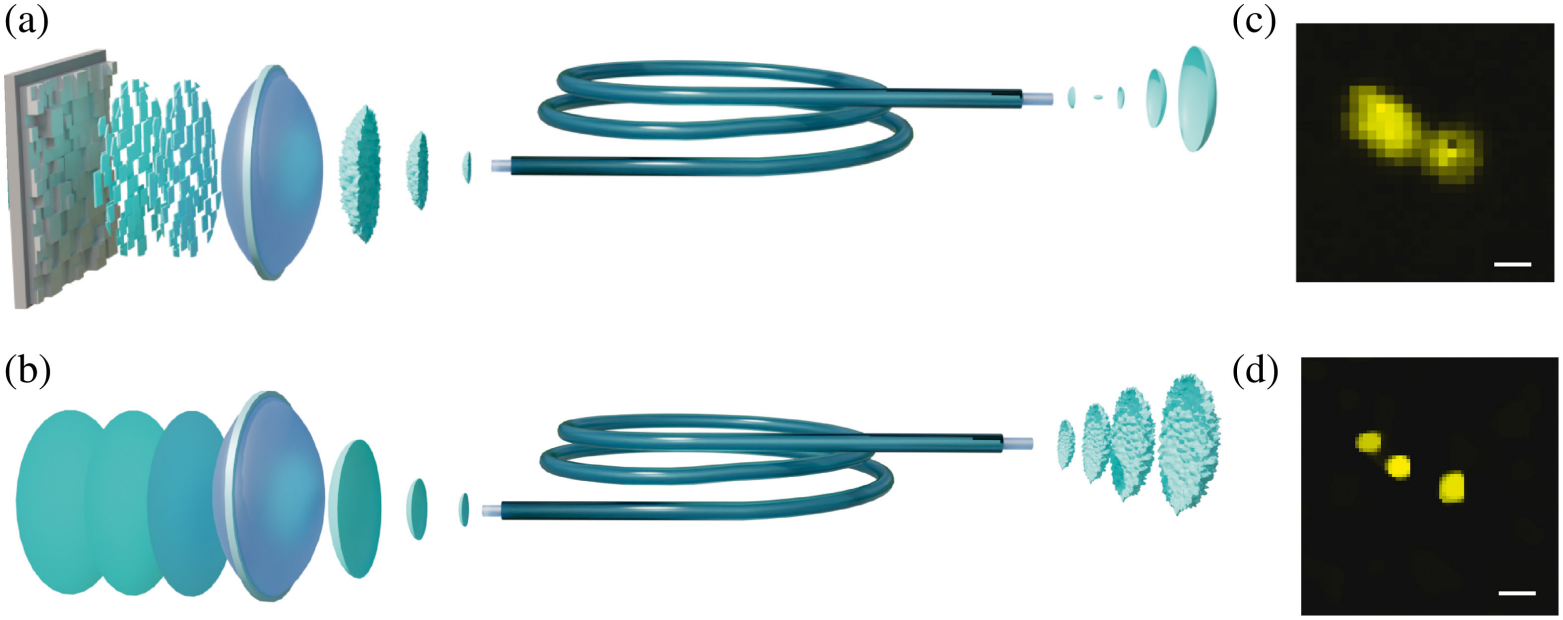
(a) Wavefront engineering on the MMF input using a spatial light modulator creates a focal spot on the fiber output facet. (b) Random patterns created within an MMF represent nearly ideal illumination for computational compressive sensing. (c), (d) Images of fluorescent beads 1.5  μm in diameter obtained through an MMF probe (NA = 0.1, pump wavelength, λ=532  nm) by (c) raster scan wavefront shaping-based endo-microscopy, which is unable to resolve beads that are <1.5  μm apart and (d) computational compressive sensing with super-resolution. The scale bars are equal to the diffraction limit λ/(2 NA)=2.66  μm. Images in (c) and (d) are adapted from Ref. 27.

An MMF is a flexible waveguide that simultaneously supports tens to thousands of guided modes propagating with different speeds.^14^ The interesting feature of light transmission through an MMF is that the process is highly complex and seemingly random but yet linear and deterministic. Coupling coherent light to an MMF results in a complex interference pattern with diffraction-limited features known as speckles but the information is only scrambled and not lost.^15^ The recent emergence of computational holography and wavefront shaping allowed for precise manipulation of the speckles by controlling the incident wavefront with a spatial light modulator.^16^^,^^17^ The capability to engineer an optical field at the MMF output to any desired pattern,^18^ e.g., a focal spot, as shown in Fig. 1(a), provides a new imaging modality: raster-scan imaging of a tissue on the MMF output facet.^19^^,^^20^ A conventional MMF can now be utilized as an ultra-thin (usually about 100  μm in diameter) aberration-free imaging probe.^21^^–^^24^ Multimode light propagation via a single core guarantees the best spatial resolution for a given footprint.^25^^,^^26^ However, it remains diffraction-limited, meaning that some subcellular structures cannot be visualized since diffraction of light blurs them to a single feature, as shown in Fig. 1(c).

In the most popular raster scan imaging approach, the ability to image a large brain region with high optical resolution is achieved at the cost of an acquisition speed. Sequential scanning of every point of interest puts a technological but still very hard limit on temporal resolution, and it is important to visualize rapid interactions between different elements of complex neuronal networks. Another issue of the state-of-the-art MMF imaging probes is its extreme sensitivity to external perturbations, such as fiber bending, movements, and temperature drifts. Even small changes in the fiber configuration destroy the imaging abilities. Therefore, despite a significant effort, use of an MMF as a high-resolution flexible probe is still far away.^15^^,^^28^^–^^32^ To fully exploit the complexity of light transport through an MMF for neuroimaging, the new technological insights are needed. Most likely it will be based on the emergence of smart and powerful computing algorithms.

## 3 Optical Imaging in the Age of Computation

Computation is becoming an integral part of imaging systems, giving rise to a new concept of designing the hardware and software components together.^33^ A joint optimization of optical setups and computational algorithms opens up new ways to overcome state-of-the-art limits of optical microscopy. While nearly all conventional signal acquisition protocols are based on the famous Nyquist–Shannon theorem (the sampling rate must be at least twice the maximum frequency of the signal), surprisingly, we rarely use all the information acquired. Storing images with lossy codecs, such as jpeg, in fact, discards the majority of acquired data. Implementing the compression already at the signal acquisition step leads to faster imaging. Computational compressive sensing facilitates signal acquisition with a large reduction in sampling for signals that have a sparse representation, vastly reducing the number of measurements beyond the Nyquist limit.^34^^,^^35^

Compressive sensing enables super-resolution imaging.^36^^,^^37^ The mechanism behind sub-diffraction compressive imaging relies on computational bandwidth interpolation. In contrast to alternative computational approaches that mainly fail because of noise, compression-based interpolation serves as an effective way for rejecting common noise types.^38^ As a result, by using an incomplete measurement set consisting of only low spatial frequency components and certain constraints (such as sparsity and continuity), the high spatial frequencies and therefore sub-diffraction features can be numerically reconstructed during post-processing. Super-resolution up to five times higher than the diffraction limit has been demonstrated in proof-of-principle experiments.^37^^,^^39^^–^^41^ Combined with structured illumination microscopy, compressive sensing helped to achieve 60-nm resolution in live-cell imaging.^42^ The spatial resolution depends on various factors, such as the measurement matrix size, the level of noise and stability, and the choice of an algorithm. A critical constraint is the sparsity of a sample. While the most natural images may not appear sparse, they still have a sparse representation, implying sparsity in a certain basis, e.g., after a wavelet transform. Therefore, identifying a proper basis and/or suitable algorithm is key for imaging biological tissues and samples that are not sparse in the regular domain.^43^

**Fig. 2:**
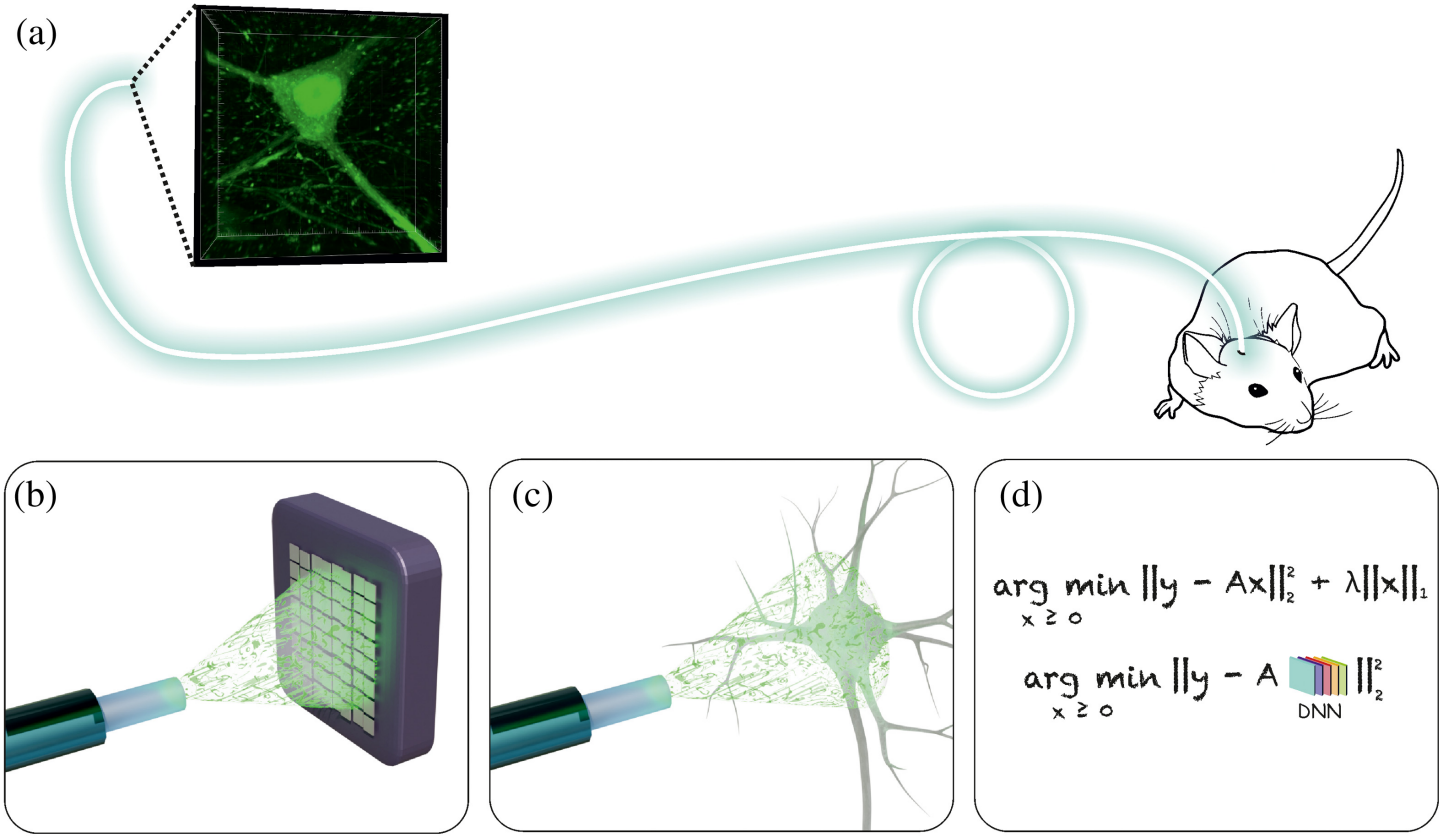
(a) Illustration of an exciting application area of computational imaging through an MMF: minimally invasive fast and super-resolution deep tissue 3D imaging in freely moving animals. (b)–(d) Three main steps of computational imaging through an MMF: (a) pre-calibration that includes recording of random patterns without the sample, (b) illuminating the sample and measuring of the total signal for each illumination pattern, and (d) computational image reconstruction using iterative algorithms of machine learning frameworks.

Compressive sensing protocols require the sampling to fulfill specific conditions to gather enough information from all parts of the object. Therefore, practical design guidelines depend on the optical system and the sample. One of the easiest and most general implementation with good reconstruction guarantee is based on randomized illumination since the random matrix is highly incoherent with any analytically fixed basis.^44^ By using an MMF, we can create the desired random illumination “for free,” as shown in Fig. 1(b) as one of the main properties of an MMF is to randomly scramble light without losing power. It makes an MMF a unique hair-thin instrument for computational imaging *in vivo* deep inside the living tissues, as shown in Fig. 2(a).

## 4 Compressive Fiber Imaging: State-of-the-Art and Outlook

Computational imaging via an MMF can be realized by illuminating the sample with a set of random patterns generated by, e.g., raster scanning of the input fiber facet with a focused spot.^45^^,^^46^ Imaging procedure consists of three main steps as shown in Figs. 2(b)–2(d). During the pre-calibration, intensity distributions of a large set of random illumination patterns are recorded, as shown in Fig. 2(b). Every image is flattened and all are assembled to two-dimensional matrix A. It is the most time-consuming experimental step, as it requires recording hundreds of images and may take from seconds to a minute depending on a camera frame rate. Fortunately, the pre-calibration is needed only once. Then, an MMF probe is inserted to the region of interest, as shown in Fig. 2(c). The sample is illuminated by the same set of patterns and the total signal (e.g., fluorescent response) for each pattern is recorded (vector, y). Compressive sensing facilitates a significant reduction of acquisition time: the lengths of y is much smaller than the lengths of flattened sample x to be reconstructed.^46^ Moreover, a camera is not necessary anymore, allowing the use of much faster detectors. The measurement rate is limited by sensitivity, field of view, and scanning speed. Utilizing a DMD (22 kHz) can provide video rate imaging at over 25 fps for up to 880 speckle patterns, well-suited for a conventional 50  μm MMF. Finally, the flattened image of a sample (x) can be reconstructed by computational algorithms that essentially perform the pseudo-inversion of the (under-determined) linear system: Ax=y, as shown in Fig. 2(d). The reconstruction speed typically ranges from seconds to minutes, depending on the number of pixels and computational power.

Computational compressive imaging, in contrast to many other super-resolution approaches, is not only integrable with an ultrathin probe, as shown in Fig. 1(d)^27^^,^^47^ but also does not require special fluorescent marks and can be used to increase resolution more than twofold beyond the diffraction limit label-free.^48^ It opens up ways to create flexible probes that do not require recalibration or access to the distal end of the fiber during imaging. Flexible probes have been demonstrated based on GRIN^49^^,^^50^ or step-index MMFs with low^51^ and high spatial resolution.^52^^,^^53^ Wavelength-dependent scattering is utilized to create a flexible probe based on a single-mode fiber.^54^ Compressive sensing improves imaging through multicore fibers by reducing acquisition time, preventing photo-bleaching, and increasing space-bandwidth product.^55^^,^^56^ Compression is also beneficial for conventional raster-scan imaging modalities helping to improve quality by harnessing “muddy” modes^57^ and to speed up pre-calibration measurements.^58^

MMFs are already used as minimally invasive probes for neuroscience and clinical applications. Wavefront shaping through an MMF probe have allowed for minimally invasive *in vivo* imaging of neurons in deep-brain regions^59^^–^^62^ and gastrointestinal imaging.^63^ Computational post-processing for a non-imaging MMF probe was used for tracking the activity of neurons.^64^ Compressive imaging through a single MMF visualized accumulation of lipofuscin in Alzheimer’s disease human brain with sub-Nyquist speed.^65^ Although many recent publications have shown the feasibility and potential of MMF imaging to become a key technology for deep-tissue brain imaging in freely moving animals, there are many challenges and associated opportunities for advancing the field. Currently, conventional diffraction limited imaging with raster scanned foci still offers better resilience to noise in a low-photon regime.^66^ Future research directions include the development of fast, precise, and robust to noise algorithms and machine learning frameworks.^67^^,^^68^

Fast high-resolution imaging of a large field of view in 3D is highly demanded.^69^ However, the transition to 3D presents several major challenges.^70^^,^^71^ The signal falls off rapidly with a distance and dense labeling may hinder deeper layers complicating the reconstruction workflow. Exploring hybrid imaging approaches, e.g., using blinking molecules, is a promising direction. Neuroscience applications require monitoring of multiple markers, therefore we need to visualize various contrast mechanisms simultaneously including quantitative label-free phase imaging.^72^ The next great challenge is to integrate different microscopy techniques into a single ultra-thin MMF probe for parallel multifunctional imaging. Finally, real-life video-rate brain imaging in freely moving animals through an ultimately thin fiber probe to be demonstrated.

To summarize, in many aspects, this new computational MMF imaging paradigm has already exceeded the current state-of-the-art. Ongoing progress in experimental design, fiber probes, and algorithms is rapidly improving both the performance and applicability. We will soon witness the computational MMF brain imaging to reach the level of readiness for technology to be transferred in the domain of *in vivo* neuroscience.

## Data Availability

Data sharing is not applicable to this article, as no new data were created.
